# Relationship between long non-coding RNA TUG1 and prognosis of patients with gastric carcinoma: A protocol for systematic review and meta-analysis

**DOI:** 10.1097/MD.0000000000023522

**Published:** 2020-12-04

**Authors:** Wei Xia, Qiang Zhang, Qian Li, Xianchun Liang

**Affiliations:** a Department of Digestive; b Department of Oncology; c Department of Hepatological Surgery, Army Medical Center of PLA, Chongqing, China.

**Keywords:** gastric carcinoma, long non-coding RNA, meta-analysis, prognosis, protocol, taurine upregulated gene

## Abstract

**Background:**

Long non-coding RNA (lncRNA) can predict the prognosis of patients with various cancers. The relationship between lncRNA taurine upregulated gene 1 (TUG1) and the prognosis of patients with gastric carcinoma still needs to be further explored. Therefore, this study attempted to explore the relationship between TUG1 and the prognosis of patients suffering from gastric carcinoma.

**Methods:**

The database was retrieved from China National Knowledge Infrastructure (CNKI), Chinese Biomedical literature Database (CBM), Chinese Scientific and Journal Database (VIP), Wan Fang database, PubMed, and EMBASE. Hazard ratios (HRs) and its 95% confidence interval (CIs) were applied to assess the prognostic effects of TUG1 on overall survival (OS). RevMan 5.3 software was adopted to perform meta-analysis.

**Results:**

The results of this meta-analysis would be submitted to peer-reviewed journals for publication.

**Conclusion:**

This review provided a comprehensive overview of the relationship between TUG1 and the prognosis of patients with gastric carcinoma, and offered recommendations for clinical practices or guidelines.

## 1 Introduction

Gastric cancer is one of the most common gastrointestinal tumors worldwide,^[1,2]^ and its mortality ranks second among all cancers.^[3]^ Most patients with advanced gastric cancer died of postoperative tumor recurrence. Gastric cancer has malignant biological characteristics, such as high incidence, easy recurrence and metastasis, and could lead to poor prognosis and low 5-year survival rate after operation.^[4]^ Therefore, to find a more effective gene target for early detection and early diagnosis of gastric cancer has become the focus of clinical researches.

In recent years, studies proved that long-chain non-coding RNA (lncRNA) can act as an oncogene or tumor suppressor gene, and participate in the occurrence of different types of tumors, including tumors in digestive system by regulating the expression of related genes.^[5]^ Recent studies revealed that taurine up-regulated gene 1 (TUG1) plays an important regulatory role in the development of bladder tumors and non-small cell lung cancer.^[6,7]^ However, the consistency and severity of the prognostic effects of TUG1 still maintain unknown. At the same time, the prognostic value of TUG1 expression in gastric cancer remains controversial. In order to verify the correlation between TUG1 and the prognosis of gastric cancer, this meta-analysis systematically integrated all published research data, so as to reveal the relationship between TUG1 and the prognostic value of gastric cancer.

## 2 Methods

### 2.1 Study registration

This meta-analysis protocol is based on Preferred Reporting Items for Systematic Reviews and meta-analysis Protocols (PRISMA-P) statement guidelines.^[8]^ The protocol of the systematic review was registered on Open Science Framework, and the registration number is DOI 10.17605/OSF.IO/KFPXZ.

### 2.2 Literature retrieval

We searched China National Knowledge Infrastructure (CNKI), Chinese Biomedical literature Database (CBM), Chinese Scientific and Journal Database (VIP), Wan Fang database, PubMed, EMBASE, and all these electronic databases, without language restrictions. PubMed searching strategy was illustrated in Table 1 in details, and other electronic databases adopted similar searching strategies.

**Table 1:** Search Strategy (PubMed).

Number	Search terms
1	Taurine upregulated gene 1[Title/Abstract]
2	TUG1[Title/Abstract]
3	or/1–2
4	Stomach Neoplasms[MeSH]
5	Cancer of Stomach[Title/Abstract]
6	Gastric Cancer[Title/Abstract]
7	Gastric Neoplasms[Title/Abstract]
8	Stomach Cancer[Title/Abstract]
9	Cancer of the Stomach[Title/Abstract]
10	Gastric Cancer, Familial Diffuse[Title/Abstract]
11	Neoplasms, Gastric[Title/Abstract]
12	Neoplasms, Stomach[Title/Abstract]
13	Cancer, Gastric[Title/Abstract]
14	Cancer, Stomach[Title/Abstract]
15	Cancers, Gastric[Title/Abstract]
16	Cancers, Stomach[Title/Abstract]
17	Gastric Cancers[Title/Abstract]
18	Gastric Neoplasm[Title/Abstract]
19	Neoplasm, Gastric[Title/Abstract]
20	Neoplasm, Stomach[Title/Abstract]
21	Stomach Cancers[Title/Abstract]
22	Stomach Neoplasm[Title/Abstract]
23	or/4-22
24	Prognos^∗^[Title/Abstract]
25	Survival[Title/Abstract]
26	or/24–25
27	3 and 23 and 26

### 2.3 Inclusion criteria for study selection

(1) Patients with gastric cancer were diagnosed by pathology or histology.
(2) TUG1was expressed in tumor tissue.
(3) Reported TUG1 survival-related data.
(4) Patients were divided into TUG1 positive and TUG1 negative.
(5) (5)Published as full-text articles.

Papers without sufficient data, literature reviews, animal studies, and other unrelated studies were excluded from the analysis.

### 2.4 Data collection and analysis

#### 2.4.1 Selection of studies

**Figure 1:**
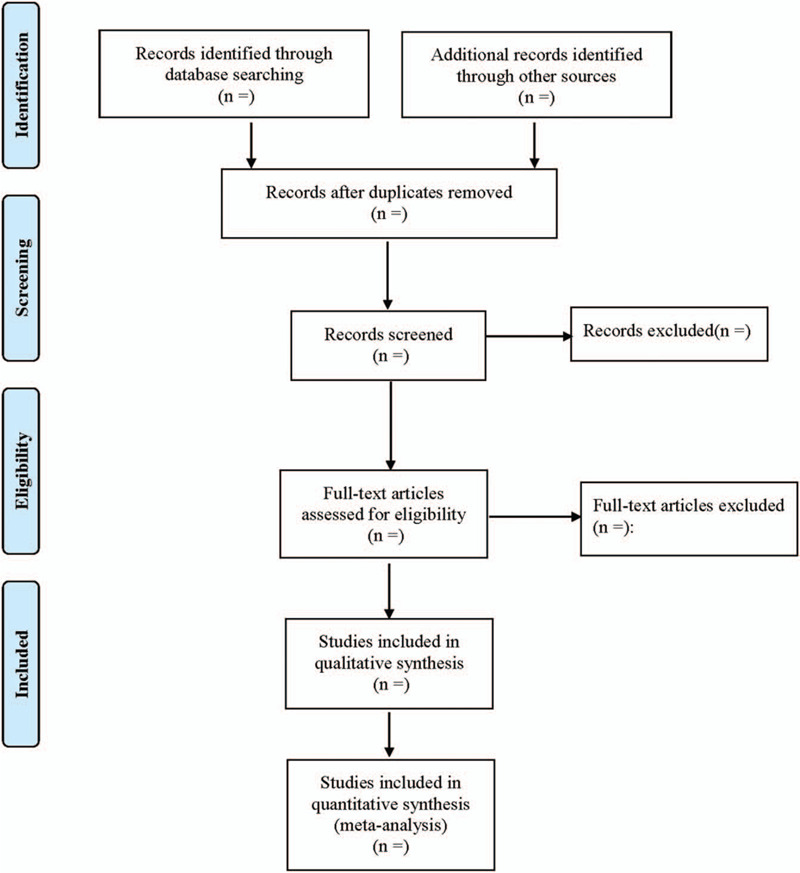
Flow diagram showing literature filtration process.

First of all, two researchers read and checked out the titles and abstracts of relevant literatures independently. After excluding the literatures that obviously do not meet the inclusion criteria, and downloading the remaining literatures for further full-text reading, the literatures that really meet the inclusion criteria were enrolled. The two researchers cross-checked the results. A self-made data extraction form was made to extract required information from the included literatures. If there exist differences, a third party would involve the discussion or consultation in this study. The flowchart of the study selection (Fig. 1) could provide a detailed description.

#### 2.4.2 Data extraction

The extracted information includes the title of the paper, name of the first author, journal, year of publication, country, race, age, sex, sample size, TUG1 detection method, TUG1 type, overall survival (OS), hazard ratio (HRs) and 95% confidence interval (CIs).

### 2.5 Literature quality evaluation

Newcastle-Ottawa quality Assessment scale (NOS) was adopted to evaluate the quality of the included studies.^[9,10]^

### 2.6 Measures of prognosis

OS was taken as prognostic outcomes, and the results were expressed as HRs with 95% CIs.

### 2.7 Management of missing data

If there exists insufficient or missing data in the literature, we would only analyze currently available data and discuss its potential value.

### 2.8 Statistical analysis

Statistical analysis was performed with RevMan 5.3 (The Nordic Cochrane Centre, The Cochrane Collaboration, 2014). HRs and 95% CIs were utilized to evaluate the relationship between LncRNA expression and OS. First of all, χ^2^ test and *I*^*2*^ test were used to test the heterogeneity of the included studies (test level α = 0.1). *I*^*2*^ ≤ 25% indicated that the heterogeneity was small, 25% < *I*^*2*^ ≤ 50% marked moderate heterogeneity, and *I*^*2*^ > 50% revealed that there was a high degree of heterogeneity among the research results. When there is no heterogeneity among the research results, the fixed effect model is adopted for meta-analysis. When there exists heterogeneity among the research results, the stability of the conclusion is verified, and the random effect model is determined for meta-analysis.

### 2.9 Additional analysis

#### 2.9.1 Subgroup analysis

According to the detection methods of TUG1, ethnicity, the source of survival data and type of TUG1, we analyzed the subgroup.

#### 2.9.2 Sensitivity analysis

Sensitivity analysis was conducted to assess the impact of individual studies on the overall merger value.

#### 2.9.3 Reporting bias

If the number of studies included in a certain outcome index was no less than 10, funnel chart could be used to evaluate publication bias.^[11,12]^

### 2.10 Ethics

Our research data was derived from published literatures, because there were no patient recruitment and personal information collection. Therefore, ethical approval was not required.

## 3 Discussion

Currently, LncRNAs is one of the hot spots in the field of tumor researches. IncRNAs have the functions of tumor-promoting genes and tumor suppressor genes. IncRNA-HAGLROS, IncRNA-AKO2339, etc. can promote the formation of gastric cancer tumors, and their high expression is a predictive molecule for the poor prognosis of gastric cancer.^[13,14]^ The expression of IncRNA-HMlincRNA717 decreases in gastric cancer, which is closely related to the recurrence of gastric cancer.^[15]^ TUG1 gene is located in the long arm of human autosome 22, zone 2, subband 2 (22q112.2), and lncRNA. TUG1 can competitively bind miRNA through competitive endogenous RNA (ceRNA) mode, and can also regulate various biological functions of cells by combining with polycomb inhibitory complex (PRC2), etc.^[16]^

Studies confirmed that lncRNA-TUGl plays a significant role in tumors developing in digestive system, urinary system, nervous system and blood system.^[17–19]^ Recently, it has been reported that TUG1 is highly expressed in gastric cancer, and its expression is related to lymph node metastasis, stage and prognosis of gastric cancer.^[20,21]^ TUG1 inhibits the miR-144/c-Met axis, and promotes the metastasis and invasion of gastric cancer cell lines.^[20]^ In addition, it can regulate the transition of cell cycle G0/G1, mainly by regulating the expression of cyclin-dependent kinase inhibitors in conjunction with PRC2, including p15, p16, p21, p27, p57, etc.^[21]^ Through meta-analysis, this study aimed to explore the role of TUG1 in gastric cancer and analyze its impact on the prognosis of gastric cancer. In order to provide a basis to judge the prognosis of gastric cancer patients, the study on whether TUG1 can be used as a more sensitive molecular marker and therapeutic target for gastric cancer should be further explored.

## Author contributions

**Conceptualization:** Xianchun Liang, Wei Xia.

**Data curation:** Qiang Zhang, Qian Li.

**Resources:** Qiang Zhang.

**Software:** Qian Li.

**Supervision:** Qiang Zhang.

**Writing – original draft:** Xianchun Liang, Wei Xia.

**Writing – review & editing:** Xianchun Liang, Wei Xia.
